# Enhanced Antibacterial Activity of Silver Nanoparticles/Halloysite Nanotubes/Graphene Nanocomposites with Sandwich-Like Structure

**DOI:** 10.1038/srep04551

**Published:** 2014-04-11

**Authors:** Liang Yu, Yatao Zhang, Bing Zhang, Jindun Liu

**Affiliations:** 1 School of Chemical Engineering and Energy, Zhengzhou University, Zhengzhou 450001, PR China

## Abstract

A sandwich-like antibacterial reagent (Ag/HNTs/rGO) was constructed through the direct growth of silver nanoparticles on the surface graphene-based HNTs nanosheets. Herein, various nanomaterials were combined by adhesion effect of DOPA after self-polymerization. Ag/HNTs/rGO posses enhanced antibacterial ability against *E. coli* and *S. aureus* compared with individual silver nanoparticles, rGO nanosheets or their nanocomposites.

Halloysite nanotubes (HNTs), a kind of aluminosilicate clay, exhibit novel physical and chemical properties due to predominantly hollow tubular structure, thereby providing opportunities for advanced applications in many fields. A significant advantage of HNTs is their easiness to obtain and low price in the comparison with other nanomaterials, like carbon nanotubes^1^,^2^,^3^,^4^. Interestingly, HNTs discovered and used by our group are provided with higher purity and quality, which have been developed into a vast of applications, for instance, phase change material^5^, enzyme immobilization^6^, adsorbents^7^ and membranes^8^.

Antibacterial reagents, including antibiotics^9^, metal ions^10^, enzymes^11^, and quaternary ammonium compounds^12^ have been extensively used to defend the public health in our daily life. However, the above materials have some drawbacks, such as, antibiotic resistance, environmental damages, relatively high cost^13^. Currently, a number of attempts have been made to develop novel, efficient and environment-friendly antibacterial materials. For instance, graphene and graphene related materials were extensively studied which exhibit strong antibacterial activity^14^,^15^,^16^. Physical damages on cell membranes are likely to occur in consequence of membrane stress induced by sharp edges of graphene nanosheets, thereby contributing to the loss of bacterial membrane integrity and the leakage of RNA^14^. In addition, silver-based nanocomposites synthetized by loading silver on the carrier were also often reported as antibacterial materials^17^,^18^. In our previous studies, copper^19^ and silver^8^,^20^ respectively were loaded on HNTs for reducing their release to fabricate antibacterial nanocomposites which showed good antibacterial performance against Gram-negative bacteria (*Escherichia coli*) and Gram-positive bacteria (*Staphylococcus aureus*). However, the antibacterial performance of individual graphene nanosheets is limited compared with silver which are easy to agglomerate contributing to the decrease of the antibacterial effects. In early approaches, graphene-AgNPs-based nanocomposites were fabricated by a number of workers which show enhanced antibacterial properties^15^,^21^,^22^,^23^,^24^. It is supposed that the direct growth of silver nanoparticles on graphene is quite capable of strengthening their antibacterial performance. Therefore, the increase of the surface area of nanocomposites may provide opportunities for the direct growth of silver nanoparticles and thus enhance the antibacterial effect deriving from the direct attachment to bacteria.

Bioadhesive proteins of marine organisms, such as mussels (Figure 1a), have attracted the public attention in the past few decades in consequence of the formation of the adhesive force on variety kinds of substrates even in wet environments^25^. The adhesive ability is attributed to L-3, 4-dihydroxyphenylala-nine (DOPA), which was discovered at a high level in adhesive proteins^26^. Lee et al.^27^ identified dopamine (Figure 1b) as a structural mimic of DOPA and demonstrated the self-polymerization of dopamine onto a wide range of inorganic or organic materials even “nonsticking surfaces” to form thin, surface-adherent polydopamine films.

Herein, HNTs were introduced to graphene-AgNPs-based nanocomposites combining the adhesive ability of DOPA to fabricate a sandwich-like nanomaterial based on the interpenetrative nanocomposites. This new approach is designed to reinforce the synergistic effects between graphene and HNTs in the interest of the direct growth of silver nanoparticles. Figure 1c illustrates the typical procedure of the construction of the sandwich-like nanomaterial. The whole procedure was accomplished under a mild environment where there are no environmentally hazardous chemicals involved in the reaction and no temperature or pressure control instrument was needed as well. DOPA deposited on the surface of HNTs through self-polymerization. The sandwich-like nanomaterial was constructed by a one-step method in which silver nanoparticles were formed via the reduction of DOPA and located on the surface of HNTs or graphene oxide (GO) nanosheets. By this means, HNTs constantly locate between GO nanosheets during the concentration and drying process. Hence, the GO nanosheets were partially reduced to graphene (rGO) and the reunion of two-dimensional graphene tends to fall off. For this reason, the specific surface area of resulted nanocomposites is inclined to increase in contrast to individual rGO or GO nanosheets in dry state. Consequently, this approach may provide an opportunity to prepare high-surface-area nanocomposites with antibacterial performance benefited from the synergistic effects from different nanomaterials.

## Results and discussion

**Figure 2:**
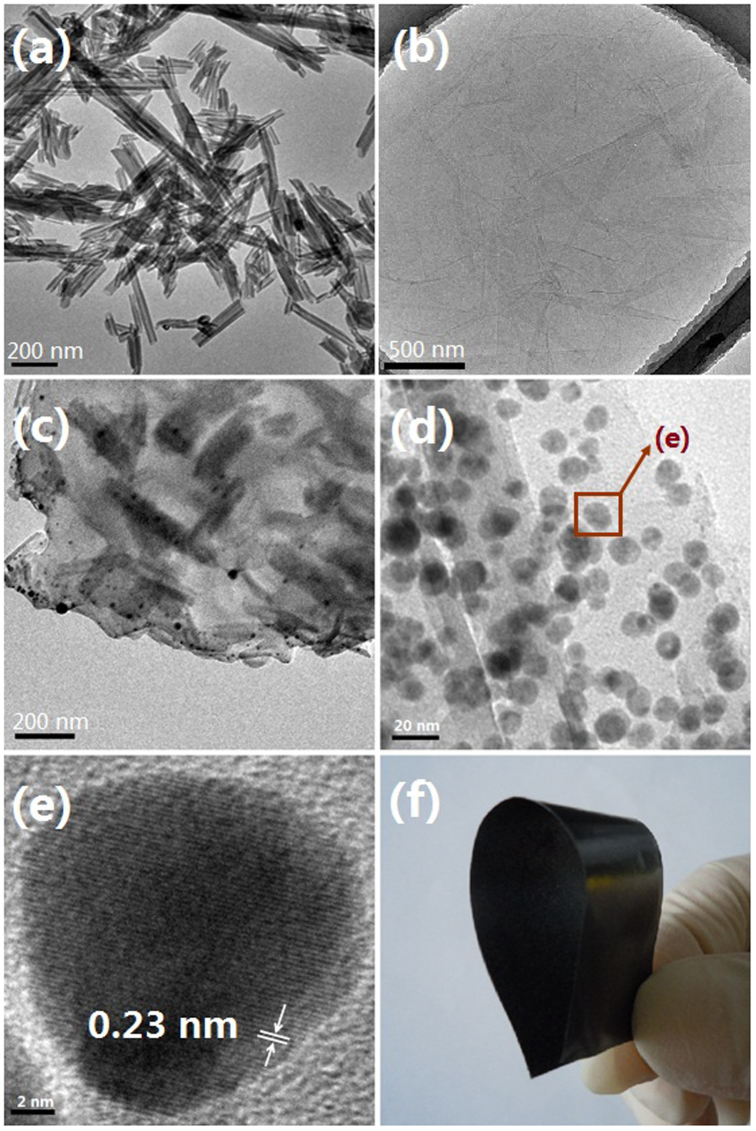
TEM images of (a) HNTs, (b) GO nanosheets and (c), (d), sandwich-like nanomaterials at different magnifications. (e) HRTEM image of single entity silver nanoparticles with fringe spacing. (f) Photograph of antibacterial film prepared from Ag/HNTs/rGO.

**Figure 3:**
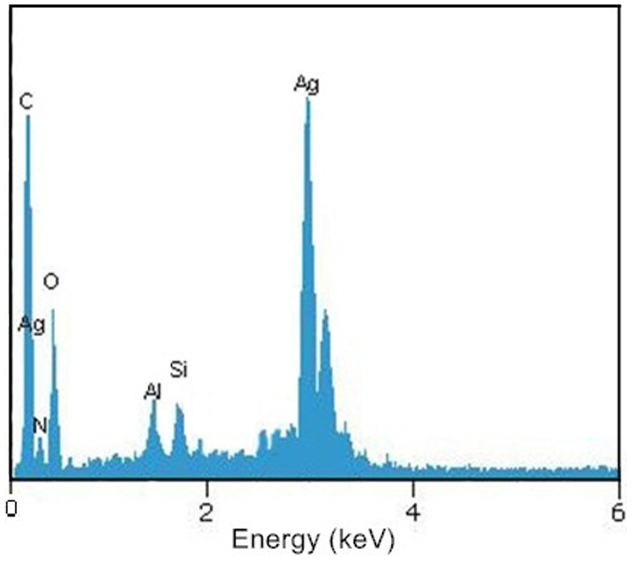
Energy dispersive spectrum of Ag/HNTs/rGO nanomaterial.

Figure 2 gives Transmission electron microscopy (TEM) images of the as-prepared sandwich-like nanomaterial (Ag/HNTs/rGO). As shown, HNTs we use (Figure 2a) have an admirable hollow tubular structure and wrinkles of GO nanosheets (Figure 2b) are clearly visible indicating the GO nanosheets are extremely thin. The resulted GO suspension is homogeneous, transparent and typically golden or brown colored. From Figure 2c and Figure 2d, it can be seen that one-dimensional HNTs intricately distribute between different GO nanosheets and silver nanoparticles directly grown on the surface of both HNTs and GO nanosheets. In Figure 2d, it is found that the silver nanoparticle size ranges from 5-15 nm. A HRTEM image of single entity silver nanoparticles is shown in Figure 2e. The crystal lattice of silver nanoparticles are resolved in most regions and the fringe spacing is found to be 0.23 nm corresponding to the (111) crystal plane^21^,^23^,^24^. In addition, energy dispersive spectrum (EDS) of Ag/HNTs/rGO is shown in Figure 3 which revealed the existence of various elements in the nanocomposites. Interestingly, a paper-like antibacterial film was prepared from Ag/HNTs/rGO by incorporation with a small amount of polyethersulfone, which shows excellent flexibility, convenience to use and may have some potential specific applications^16^.

**Figure 4:**
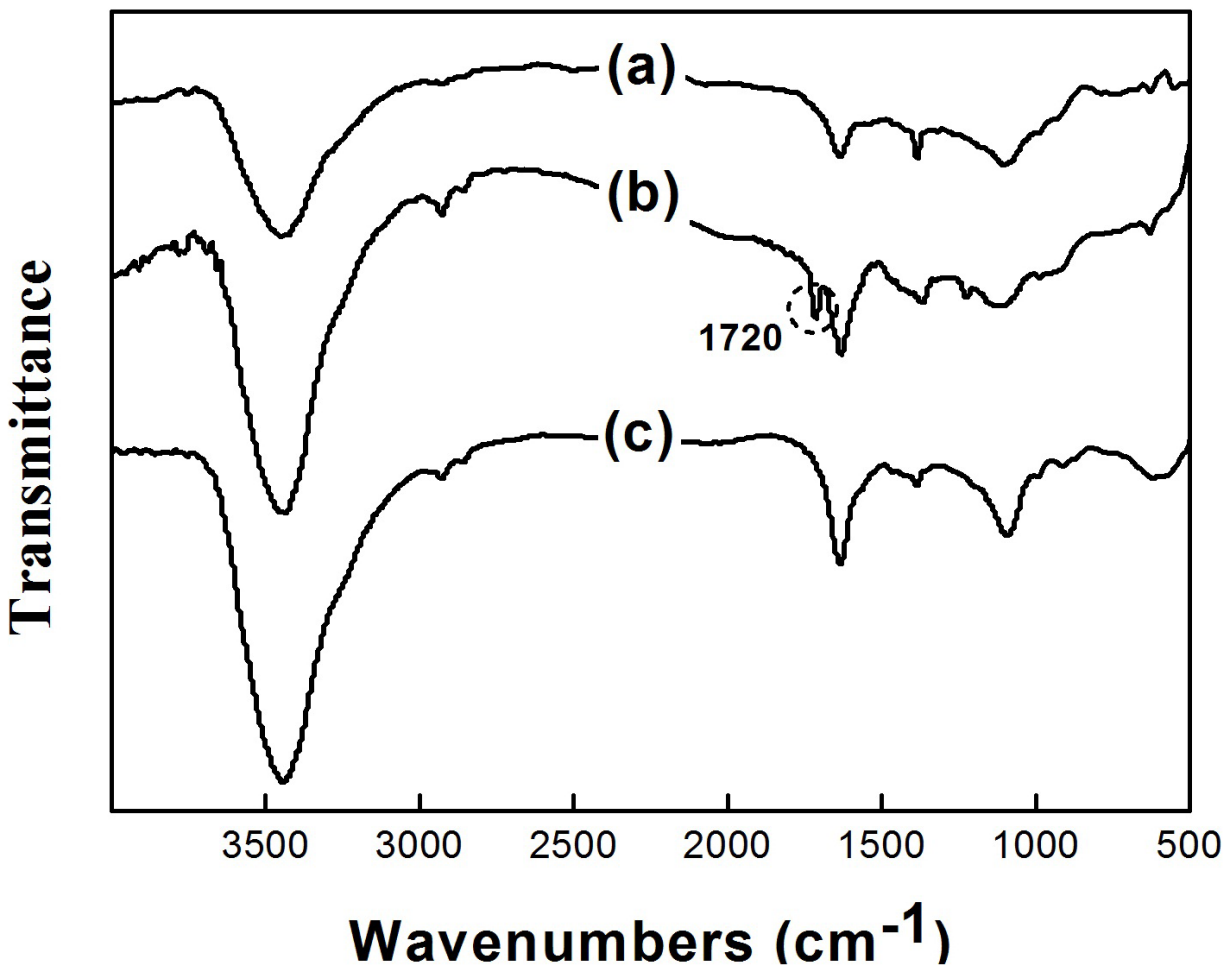
FTIR spectra of (a) natural graphite, (b) GO and (c) Ag/HNTs/rGO.

Fourier transform infrared spectroscopy (FTIR) of Ag/HNTs/rGO was performed to justify whether GO nanosheets was reduced to rGO during the construction process of the sandwich-like nanomaterial. It can be seen from Figure 4, the curve of GO represented a considerable absorption peak around 1720 cm^−1^, which ascribes to C = O stretching vibrations in the carboxyl group of GO after the oxidation of natural graphite. However, after the construction of Ag/HNTs/rGO, the characteristic peak around 1720 cm^−1^ vanished and the whole curve of Ag/HNTs/rGO was almost identical to that of natural graphite. The two small peaks around 2930 cm^−1^ and 2840 cm^−1^ of Ag/HNTs/rGO are supposed to be in the consequence of the partial destruction of carbon skeleton of natural graphite during the oxidation process, which tends not to be restored thoroughly by reduction. These results indicated that GO nanosheets were reduced to rGO by DOPA during the fabrication process of Ag/HNTs/rGO.

To investigate the surface area change of Ag/HNTs/rGO, the specific surface area (BET) was measured using the adsorption of N_2_ at the temperature of liquid nitrogen. The specific surface area (SBET) for Ag/HNTs/rGO showed an obvious enhancement with a value 287.10 m^2^/g. Nevertheless, the SBET value for Ag/HNTs and Ag/rGO were 59.6 m^2^/g and 128.92 m^2^/g, respectively. That is to say, a high-surface-area substrate was synthetized as our initial design.

The antibacterial properties of Ag/HNTs/rGO was studied against Gram negative bacterial strains, *E. coli* and Gram positive bacterial strains, *S. aureus* as bacterium models. Minimal inhibitory concentration (MIC) was observed by the tube double dilution method to evaluate the effectiveness of as-prepared nanomaterials. In the tube double dilution method, MIC is defined as the lowest concentration (in μg/ml) of the antimicrobial agent that prevents visible growth of a microorganism under defined conditions. The MIC results of the sandwich-like nanomaterial, Ag/HNTs/rGO and the controls including Ag, Ag/HNTs, rGO/Ag is shown in Table 1. Note that all the mentioned Ag is at the nanoscale. The MIC of all the prepared nanomaterials followed the order: rGO > Ag > Ag/HNTs > Ag/rGO > Ag/HNTs/rGO. Compared to all the control groups, Ag/HNTs/rGO exhibited a lowest MIC of 2 μg/ml indicating excellent antibacterial effect. The phenomenon may attribute to the synergistic antibacterial effect from Ag and rGO. Furthermore, the enhanced surface area of Ag/HNTs/rGO is another important factor for the excellent antibacterial effect which may reduce the reunion of silver nanoparticles. Generally, the aggregation of antibacterial nanomaterial would greatly decrease their antibacterial activities^22^. In this work, silver nanoparticles were loaded onto the surface of the sandwich-like nanomaterial and in-situ reduced by DOPA. Thus, the aggregation of silver nanoparticles was effectively decreased.

**Figure 5:**
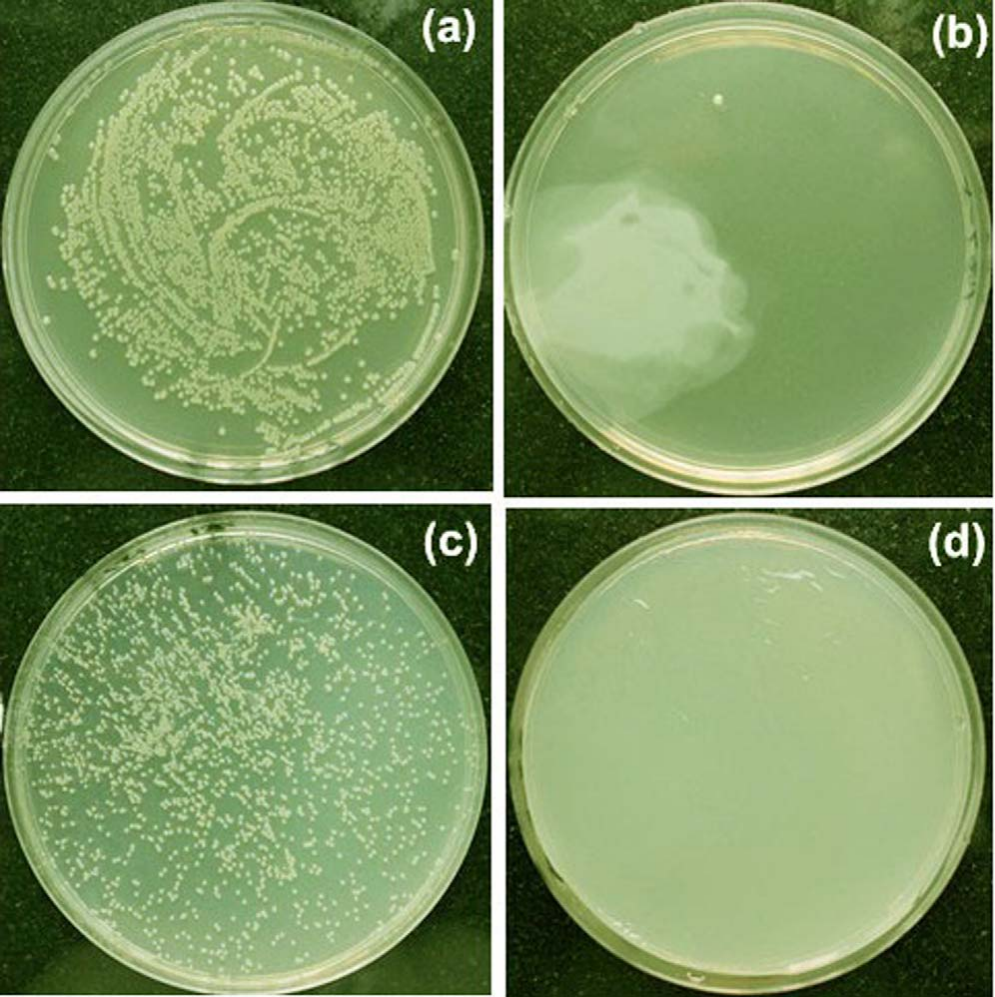
Photographs of bacterial colonies formed by (a), (b) *E. coli* and (c), (d) *S. aureus*: (a), (c) control groups, (b), (d) treated with Ag/HNTs/rGO.

Additionally, the sandwich-like nanomaterial showed excellent antibacterial performance through bacteriostasis rate test. It can be seen in Figure 5 that colonies of *E. coli* or *S. aureus* without treated by Ag/HNTs/rGO are observed as small white dots and almost fill the whole plate as shown in Figure 5a, c. In the comparison, in Figure 5b, d few colonies can be visibly observed on the plates treated by Ag/HNTs/rGO. The superior bacteriostasis rate nearing 100% against both *E. coli* and *S. aureus* demonstrate the effectiveness and popularity of the antibacterial properties of Ag/HNTs/rGO.

**Figure 6:**
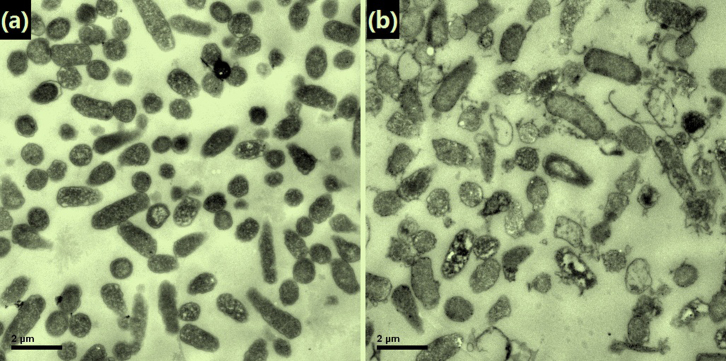
TEM images of *E. coli* cells before (a) and after (b) treated with Ag/HNTs/rGO.

The morphology changes of *E. coli* cells were further analyzed by using TEM. As shown in Figure 6a, the original *E. coli* cells were evenly fuscous with well-defined membranes. However, after treated with Ag/HNTs/rGO for 16 h, the *E. coli* cells were ruptured, pale along with the release of cytoplasm and a large portion of the cells was decomposed as can be seen in Figure 6b. Such irreversible cellular damage demonstrates the effectiveness of the antibacterial properties of Ag/HNTs/rGO.

**Figure 7:**
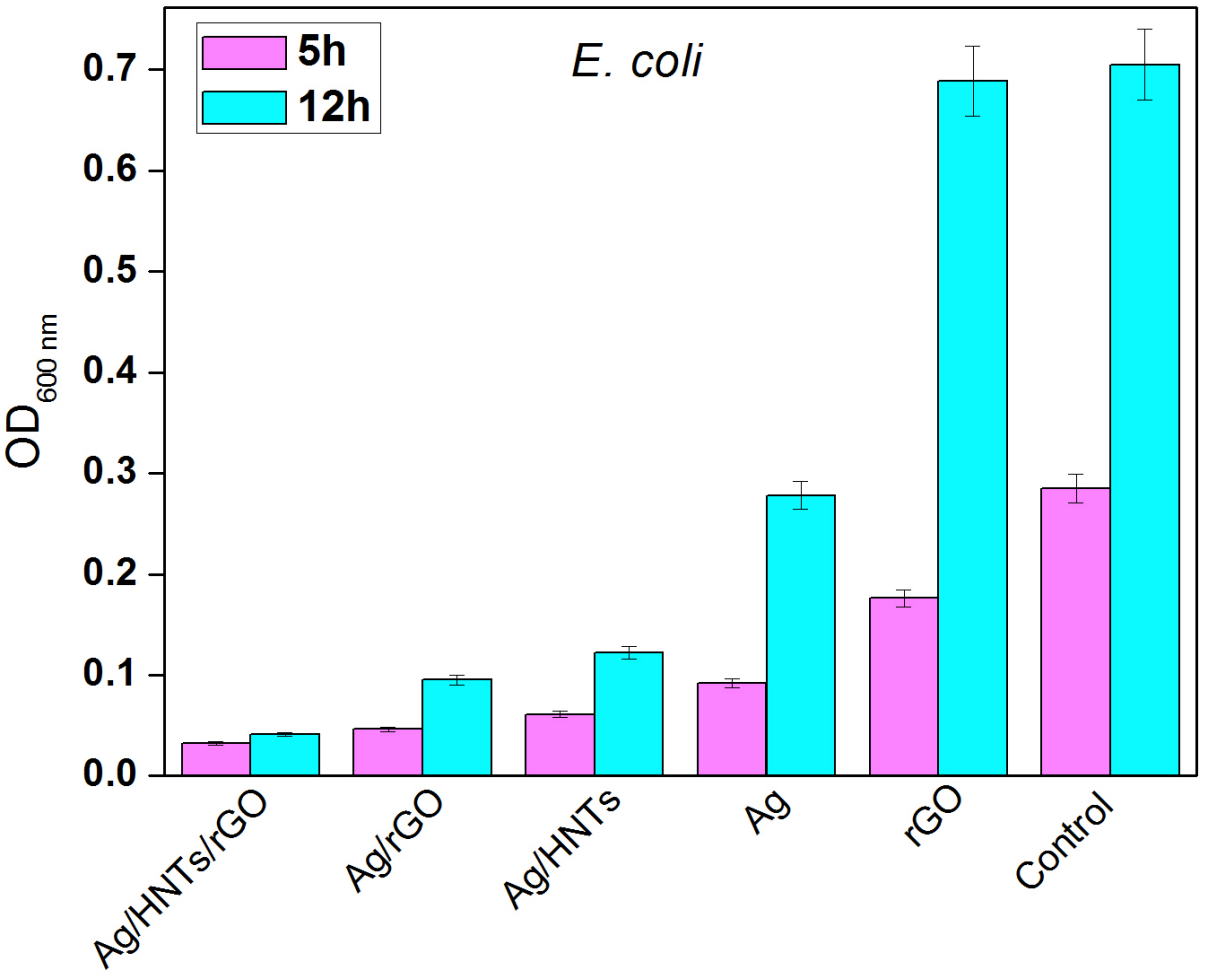
Optical density at 600 nm (OD_600_) of bacterial suspension treated with different reagents for 5 and 12 h.

The optical density at 600 nm (OD_600_) of *E. coli* suspension treated with different prepared reagents in test tubes for 5 h and 12 h were determined, which is a widely used method to determine the growth of bacteria for the assessment of antibacterial abilities^28^,^29^. Generally, the lower optical density at 600 nm of bacteria suspension after cultivation for a definite time means the better antibacterial ability of antibacterial reagent. As can be seen from Figure 7, for different prepared antibacterial reagent, the OD_600_ values followed the order: control > rGO > Ag > Ag/HNTs > Ag/rGO > Ag/HNTs/rGO, which is almost the same with the MIC of them. Silver nanoparticles which grow directly from Ag/HNTs, Ag/rGO, and Ag/HNTs/rGO showed lower OD_600_ after both 5 h and 12 h cultivation. This phenomenon can be on account of the decrease of conglomeration of silver nanoparticles. Additionally, each group of data presents a good stability with a very minor anova. It is important to emphasize that the sandwich-like nanomaterial, Ag/HNTs/rGO, showed the lowest OD_600_ especially after 12 h cultivation in contrast to other antibacterial reagents. These results indicated that Ag/HNTs/rGO possesses a better ability in resisting bacteria growth agreeing strongly with the MIC results, which probably benefits from enhanced surface area of Ag/HNTs/rGO.

In summary, a sandwich-like antibacterial reagent was constructed through the direct growth of silver nanoparticles on the surface graphene-HNTs-based nanocomposites. Different nanomaterials were combined by adhesion effect of DOPA after self-polymerization. A series of experiments were performed and the results showed that Ag/HNTs/rGO has enhanced antibacterial ability against *E. coli* and *S. aureus* compared with individual silver nanoparticles, rGO nanosheets or their nanocomposites. It is anticipated that the sandwich-like antibacterial nanomaterial may be used in some potential antibacterial applications, antibacterial ultrafiltration membranes used for water treatment^30^.

## Methods

### Materials

Graphite powders (spectral pure) were purchased from Sinopharm Chemical Reagent Co., Ltd., and were used as received. Halloysite clay from Henan Province (China) was milled and sieved to obtain halloysite nanotubes (HNTs). Tris (hydroxymethyl) aminomethane (AP) and dopamine (AP) were purchased from Sigma Aldrich. Polyethersulfone (PES, Ultrason E6020P with Mw = 58 kDa) was obtained from BASF, Germany. All the other chemicals (analytical grade) were obtained from Tianjin Kermel Chemical Reagent Co., Ltd., China, and were used without further purification. The used water is deionized water.

### Fabrication of sandwich-like nanomaterial (Ag/HNTs/rGO)

GO nanosheets were prepared by oxidizing natural graphite based on an improved method^31^ and was used in aqueous solution. Dopamine, 200 mg, was dissolved in a 100 mL Tris-HCl solution. HNTs, 0.3 g, were dispersed in the above solution by ultrasonic to react for 16 h under stirring at ambient condition. Subsequently, the treated particles were washed by deionized water thoroughly and dispersed in a 250 ml GO aqueous solution (2 mg/mL). Then, Tollens' reagent was added to the mixed solution to react overnight at room temperature under stirring. Finally, the product was collected by centrifugation and washed with deionized water and ethanol, respectively. The wet product was vacuum dried at room temperature for 24 h.

### Fabrication of Ag/HNTs/rGO film

As prepared Ag/HNTs/rGO particles, 0.3 g, were added in 5 mL N, N-Dimethylacetamide (DMAc) and dispersed thoroughly by stirring. Then PES, 0.9 g, was added under stirring and the stirring process continued at least 24 h. After PES completely dissolved in the solution, the mixture was poured into a home-made glass groove with about 3 mm deepness. The solvent was evaporated thoroughly under an open environment at room temperature and the Ag/HNTs/rGO film was peeled off from the glass carefully. The resulted Ag/HNTs/rGO has a 20 × 60 mm^2^ area with about 1 mm thickness.

### Characterization

FTIR spectra were performed at 2 cm^−1^ resolution with Thermo Nicolet IR 200 spectroscope (Thermo Nicolet Corporation, USA). Typically, 64 scans were signal-averaged to reduce spectral noise. The spectra were recorded in the 400–4000 cm^−1^ range using KBr pellets. A FEI model TECNAI G^2^ transmission electron microscope (FEI, USA) was used to study the morphology of Ag/HNTs/rGO. The samples were dispersed in solvent with the aid of ultrasound. The suspended particles were transferred to a copper grid (400 meshes) coated with a strong carbon film and dried. Energy Dispersive System (EDS) was carried out in a JEOL JSM-7500F FE-SEM with samples sputtered with gold.

### Bacterial killing experiments

Minimal inhibitory concentration (MIC): Serial dilutions of the antibacterial materials were performed in Mueller-Hinton broth which were inoculated with 5 × 10^5^ CFU/mL of bacteria and incubated overnight at 37°C for 8 h. Growth of the cells was determined by observing the turbidity of the culture. The lowest concentration, at which no visual turbidity could be observed, represented the MIC of the antibacterial material.

#### Bacteriostasis rate

A 100 μL of the above bacterial suspension after being diluted 10^5^ times was cultured on an agar plate. After incubated at 37°C for 16 h, the numbers of colonies were counted and the bacteriostasis rate was determined by dividing the number of colony-forming units (CFU) which are killed by antibacterial reagent by the number of CFU of the control group.

Transmission Electron Microscopic (TEM) Measurements: *E. coli* cells treated with Ag/HNTs/rGO solution for 30 min were fixed with 2.5% glutaraldehyde. The cells were washed with PBS and then postfixed with 1% aqueous OsO_4_ (Fluka) for 1 h and washed again twice with PBS. The cells then were dehydrated through ethanol series (70% for 15 min, 90% for 15 min, and 100% for 15 min twice) and embedded in Epon/Araldite resin (polymerization at 65°C for 15 h). Thin sections (90 nm) containing the cells were placed on the grids and stained for 1 min each with 4% uranyl acetate (1:1 acetone/water) and 0.2% Raynolds lead citrate (water), air dried, and examined under a transmission electron microscope (FEI, USA).

## Author Contributions

Y.T.Z. and B.Z. conceived and designed the study. Y.T.Z. and J.D.L. wrote the manuscript. L.Y. performed the experiments. All the authors contributed to discussion and reviewed the manuscript.

## Figures and Tables

**Figure 1:**
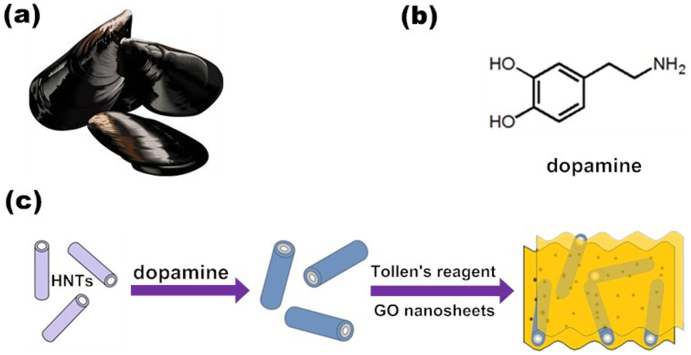
Fabrication process for sandwich-Like, silver nanoparticles/halloysite nanotubes/graphene nanocomposites (Ag/HNTs/rGO).

**Table 1:** MIC of as-synthesized different nanomaterials to *E. coli* (μg/ml)

rGO	Ag	Ag/HNTs	Ag/rGO	Ag/HNTs/rGO
1024	64	32	16	2
